# Accelerated directed evolution of dye-decolorizing peroxidase using a bacterial extracellular protein secretion system (BENNY)

**DOI:** 10.1186/s40643-019-0255-7

**Published:** 2019-05-31

**Authors:** Abdulrahman H. A. Alessa, Kang Lan Tee, David Gonzalez-Perez, Hossam E. M. Omar Ali, Caroline A. Evans, Alex Trevaskis, Jian-He Xu, Tuck Seng Wong

**Affiliations:** 1 Department of Chemical & Biological Engineering and Advanced Biomanufacturing Centre, University of Sheffield, Sir Robert Hadfield Building, Mappin Street, Sheffield, S1 3JD UK; 2 Laboratory of Biocatalysis and Bioprocessing, State Key Laboratory of Bioreactor Engineering, East China University of Science and Technology, 130 Meilong Road, Shanghai, 200237 People’s Republic of China

**Keywords:** Directed evolution, Extracellular protein secretion, Dye-decolorizing peroxidase, Osmotically-inducible protein Y, Hydrogen peroxide tolerance

## Abstract

**Background:**

Dye-decolorizing peroxidases (DyPs) are haem-containing peroxidases that show great promises in industrial biocatalysis and lignocellulosic degradation. Through the use of *Escherichia coli* osmotically-inducible protein Y (OsmY) as a bacterial extracellular protein secretion system (BENNY), we successfully developed a streamlined directed evolution workflow to accelerate the protein engineering of DyP4 from *Pleurotus ostreatus* strain PC15.

**Result:**

After 3 rounds of random mutagenesis with error-prone polymerase chain reaction (epPCR) and 1 round of saturation mutagenesis, we obtained 4D4 variant (I56V, K109R, N227S and N312S) that displays multiple desirable phenotypes, including higher protein yield and secretion, higher specific activity (2.7-fold improvement in *k*_cat_/*K*_m_) and higher H_2_O_2_ tolerance (sevenfold improvement based on IC_50_).

**Conclusion:**

To our best knowledge, this is the first report of applying OsmY to simplify the directed evolution workflow and to direct the extracellular secretion of a haem protein such as DyP4.
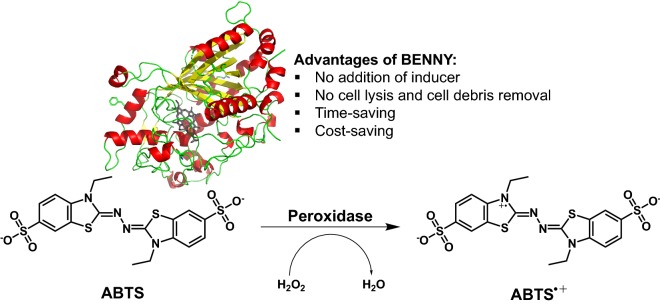

**Electronic supplementary material:**

The online version of this article (10.1186/s40643-019-0255-7) contains supplementary material, which is available to authorized users.

## Introduction

Dye-decolorizing peroxidases (DyPs; PF04261; EC 1.11.1.19) comprise a recently described family of haem peroxidase enzymes, which is unrelated to the superfamilies of plant and animal peroxidases (Martinez et al. 2017). According to the RedoxiBase [http://peroxibase.toulouse.inra.fr/; accessed on 07/04/19, (Fawal et al. 2013)] a total of 237 DyPs have been identified in the genomes of fungi, bacteria and archaea. Although their physiological functions are yet to be fully elucidated, DyPs have several characteristics that distinguish them from all other peroxidases. They exhibit low sequence similarity to the classical fungal peroxidases, such as lignin peroxidase (LiP; EC 1.11.1.14) and manganese peroxidase (MnP; EC 1.11.1.13). Structural characterization of DyPs revealed the presence of a two-domain, α + β ferredoxin-like fold that is distinct from the all α-helical fold of the other peroxidase superfamilies (Singh and Eltis 2015). Initial structure- and sequence-based alignments identified four phylogenetically distinct classes of DyPs (A to D). Classes A to C predominantly contain bacterial sequences while class D is mostly represented by fungal DyPs. DyPs are bifunctional enzymes displaying not only oxidative activity but also hydrolytic activity (Hofrichter et al. 2010). They show a particularly wide substrate range and function well under much lower pH conditions compared to other plant peroxidases. They are also able to oxidize a variety of organic compounds of which some are poorly converted by established peroxidases, including dyes (e.g. anthraquinone-based industrial dyes), β-carotene and aromatic sulphides. Accumulating evidence shows that DyPs play a key role in lignin degradation. Owing to these unique properties, DyPs are potential candidates for a variety of biotechnological applications.

In this article, we enhanced the hydrogen peroxide (H_2_O_2_) tolerance of dye-decolorizing peroxidase 4 (DyP4) from *Pleurotus ostreatus* strain PC15 (oyster mushroom) using directed evolution. DyP4 was the first reported fungal DyP capable of oxidizing manganese (II) (Fernandez-Fueyo et al. 2015). Additionally, it oxidizes both low and high redox-potential dyes. It also displays high thermal and pH stability. DyP4 was detected in the secretome of *P. ostreatus* grown on different lignocellulosic substrates, suggesting that the generation of Mn^3+^ oxidizers plays a role in the *P. ostreatus* white-rot lifestyle.

To accelerate DyP4 evolution, we used a bacterial extracellular protein secretion system (BENNY) based on the *Escherichia coli* osmotically-inducible protein Y (OsmY). OsmY was originally identified as a naturally excreted protein in a systematic proteomic analysis of the extracellular proteome of *E. coli* BL21 (DE3) (Qian et al. 2008). It was subsequently used as a fusion partner to direct the extracellular secretion of various recombinant proteins expressed in *E. coli*, including endoglucanase (Gupta et al. 2013), β-glucosidase (Gupta et al. 2013), xylanase (Zheng et al. 2012; Le and Wang 2014), xylosidase (Zheng et al. 2012), single-chain antibody (Cheng et al. 2014) and various human proteins (Kotzsch et al. 2011). To our best knowledge, this is the first report of applying OsmY to streamline directed evolution workflow and to direct extracellular secretion of a haem protein such as DyP4.

## Materials and methods

### Materials

Chemicals were purchased from Sigma-Aldrich (Dorset, UK) and ForMedium (Norfolk, UK). DNA modifying enzymes, deoxyribonucleotides and DNA ladders were purchased from New England Biolabs (Hitchin, UK), Thermo Fisher Scientific (Loughborough, UK) and Agilent Technologies (Cheadle, UK). Nucleic acid purification kits were purchased from Qiagen (Manchester, UK), Machery-Nagel (Düren, Germany) and Omega Bio-tek (Norcross, USA). All oligonucleotides were synthesized by Eurofins Genomics (Ebersberg, Germany) and summarized in Table 1.

**Table 1:** Oligonucleotides used in this study

Oligonucleotide	Applications	DNA sequence (5′–3′)
NdeI-DyP4-Fwd	Cloning	TATACATATGATGACCACCCCGGCGCCGCCGCTG
DyP4-Rev	Cloning, epPCR, SM	ATGCGAATTCTTACGCGCTGATCGGCGCTTGGCTGTGC
BamHI-DyP4-Fwd	epPCR, SM	GATCGGATCCATGACCACCCCGGCGCCGCCGCTGG
M43L-F	SDM	AAAGCGAACCTGGCGCACTTCATCCCGCACATTAAGACCAGCGCGG
M43L-R	SDM	GAAGTGCGCCAGGTTCGCTTTAAATTGATCAACGTTGGTCACGTCG
M77L-F	SDM	CTGGTGCCGCTGGCGGCGGTGAACGTTAGCTTTAGCCACCTGGGCC
M77L-R	SDM	CACCGCCGCCAGCGGCACCAGACCCGGTTTCTTCTGACGTTTGTGT
M253L-F	SDM	CTGTTCCAACTGGTGCCGGAGTTTGACGATTTCCTGGAAAGCAACC
M253L-R	SDM	CTCCGGCACCAGTTGGAACAGGTAACGGAAGGTCAGAAAGCTACCA
M253F-F	SDM	CTGTTCCAATTTGTGCCGGAGTTTGACGATTTCCTGGAAAGCAACC
M253F-R	SDM	CTCCGGCACAAATTGGAACAGGTAACGGAAGGTCAGAAAGCTACCA
NOP-312N-F	SM	TGCGCAGCGTNNKAACAAGTTTGACTTC
NOP-312N-R	SM	TCCGCCGCCAGTTTCGGATCGTCCT
4P-56N-F	SM	ACCAGCGCGGGCNNKATTAAAGACCGTGAG
4P-56N-F	SM	TTTAATMNNGCCCGCGCTGGTCTTAATGTG
4P-109N-F	SM	ACCGGCCAGCGTNNKGACGCGGAGATTCTG
4P-109N-R	SM	CGCGTCMNNACGCTGGCCGGTGGTGAACGC
4P-227N-F	SM	CTGGCGAAGGAGNNKGGTGACAGCCGTGCG
4P-227N-R	SM	GTCACCMNNCTCCTTCGCCAGAATGAAACC
4P-306N-F	SM	GATCCGAAACTGNNKGCGGATGCGCAGCGT
4P-306N-R	SM	ATCCGCMNNCAGTTTCGGATCGTCCTTCAG
4P-374N-F	SM	ACCAGCCAAGAANNKCACGACAAGAAAACC
4P-374N-R	SM	GTCGTGMNNTTCTTGGCTGGTCACTTCCGG

*epPCR* error-prone polymerase chain reaction, *SDM* site-directed mutagenesis, *SM* saturation mutagenesis

### Strains

*Escherichia coli* DH5α was used for all molecular cloning, plasmid propagation and maintenance. *E. coli* BL21 (DE3) (Merck; Darmstadt, Germany) was used for DyP4 and OsmY-DyP4 protein expression.

### Molecular cloning of DyP4 and OsmY-DyP4

The DNA sequence encoding both the *E. coli* osmotically-inducible protein Y (OsmY; GenBank: AUY30809.1) and the *Pleurotus ostreatus* strain PC15dye-decolorizing peroxidase 4 (DyP4; GenBank: KP973936.1) was codon-optimized for protein expression in *E. coli* and synthesized by GenScript (Piscataway, USA). The gene (Additional file 1: Figure S1) was cloned into pET-24a(+) vector (Merck; Darmstadt, Germany) using *Nde*I and *Eco*RI sites, and the resulting plasmid [pET-24a(+)-OsmY-DyP4; Additional file 1: Figure S2] was used for protein engineering in this study. To create pET-24a(+)-DyP4 plasmid, DyP4 gene was amplified with NdeI-DyP4-Fwd and DyP4-Rev primers, digested with *Nde*I and *Eco*RI and cloned into pET-24a(+) vector.

### Random mutagenesis by epPCR

Three error-prone polymerase chain reaction (epPCR) conditions were used in this study: high (H), medium (M) and low (L) error rates. For epPCR of high error rate, the 50-μL PCR mixture contained 1× standard Taq reaction buffer (Mg-free), 7 mM MgCl_2_, 0.05 mM MnCl_2_, 0.2 mM of each dNTP, 20 pmol BamHI-DyP4-Fwd primer, 20 pmol DyP4-Rev primer, 50 ng pET-24a(+)-OsmY-DyP4 and 1.25 U Taq DNA polymerase (New England Biolabs). For epPCR of medium error rate, the 50-μL PCR mixture contained 1× standard Taq reaction buffer (Mg-free), 7 mM MgCl_2_, 0.2 mM dATP, 0.2 mM dGTP, 1 mM dTTP, 1 mM dCTP, 20 pmol BamHI-DyP4-Fwd primer, 20 pmol DyP4-Rev primer, 50 ng pET-24a(+)-OsmY-DyP4 and 1.25 U Taq DNA polymerase (New England Biolabs). For epPCR of low error rate, the 50-μL PCR mixture contained 1× standard Taq reaction buffer (Mg-free), 1.5 mM MgCl_2_, 0.01 mM MnCl_2_, 0.3 mM of each dNTP, 4.5 pmol BamHI-DyP4-Fwd primer, 4.5 pmol DyP4-Rev primer, 3.5 ng pET-24a(+)-OsmY-DyP4 and 1.25 U Taq DNA polymerase (New England Biolabs). The PCR mixtures were thermocycled using the following conditions: (i) 30 s initial denaturation at 95 °C, (ii) 30 cycles of 20 s denaturation at 95 °C, 30 s annealing at 68 °C and 1 min 30 s extension at 68 °C and (iii) 5 min final extension at 68 °C. PCR products were either purified by gel extraction (high and medium error rate) or PCR purification following *Dpn*I digestion (low error rate). After restrictive digestion with *Bam*HI and *Eco*RI, the PCR products were cloned into pET-24a(+)-OsmY vector. The recombinant plasmids were subsequently electroporated into *E. coli* BL21 (DE3) to create OsmY-DyP4 mutant libraries.

### Cultivation and protein expression in 96-well microtitre plates

Individual colonies were picked manually using sterile toothpicks into 96-well microtitre plates, with each well containing 150 μL 2× TY medium (16 g/L tryptone, 10 g/L yeast extract and 5 g/L NaCl) supplemented with 50 μg/mL kanamycin. Wells B2, E6 and G11 were inoculated with either wildtype (WT) or parental strain as internal control. Plates were covered with lids, sealed and cultivated at 30 °C for 24 h. Following cultivation, 100 μL of 50% (v/v) glycerol solution was added to each well, and these master plates were stored at − 80 °C.

To prepare pre-culture for protein expression, master plates were replicated using a pin replicator into fresh 96-well microtitre plates, with each well containing 150 μL 2× TY medium supplemented with 50 μg/mL kanamycin. These pre-culture plates were grown at 30 °C for 18 h, before being used to inoculate fresh 96-well microtitre plates, with each well containing 150 μL 2× TY-based auto induction medium [AIM; 16 g/L tryptone, 10 g/L yeast extract, 3.3 g/L (NH_4_)_2_SO_4_, 6.8 g/L KH_2_PO_4_, 7.1 g/L Na_2_HPO_4_, 0.5 g/L glucose, 2.0 g/L α-lactose and 0.15 g/L MgSO_4_] supplemented with 50 μg/mL kanamycin. After cultivation at 30 °C for 24 h, the plates were centrifuged at 4000 rpm (eq. 2342 g) for 10 min. The spent medium containing secreted OsmY-DyP4 was used for high-throughput screening (HTS).

Abgene 96-well polypropylene storage microplates (Thermo Fisher Scientific; AB0796) and Abgene polypropylene plate covers (Thermo Fisher Scientific; AB0755) were used in preparing master plates, pre-culture plates and protein expression plates. All plate cultivations were conducted in Titramax1000 plate shaker coupled to an Incubator 1000 heating module (Heidolph Instruments; Essex, UK) using a shaking speed of 1050 rpm.

### Screening for higher H_2_O_2_ tolerance

Flat-bottom clear 96-well polystyrene microplates (Greiner Bio-One; 655161) were used for screening. Twenty microlitre of spent medium was transferred to 96-well microtitre plate, with each well containing 150 μL of 10 mM 2,2′-azino-bis(3-ethylbenzothiazoline-6-sulphonic acid) (ABTS) prepared in 0.1 M citrate–0.2 M Na_2_HPO_4_ pH 3.4 buffer solution. The mixture was shaken for 2 min before reaction was initiated by adding 50 μL of 17.5 mM H_2_O_2_ solution. Absorbance at 405 nm was recorded with Multiskan FC microplate photometer (Thermo Fisher Scientific), after a 2-min reaction with shaking. All solutions were freshly prepared prior to screening. All shaking steps were conducted in Titramax 1000 (Heidolph Instruments) using a shaking speed of 1050 rpm.

### Site-directed mutagenesis and saturation mutagenesis

Mutagenic primers (Table 1), PCR mixtures and PCR conditions for all site-directed mutagenesis and saturation mutagenesis studies were designed using OneClick programme, which is publicly accessible via the web-link: http://tucksengwong.staff.shef.ac.uk/OneClick/ (Warburton et al. 2015).

All methionine-substituted variants were created using pET-24a(+)-DyP4 as template. To construct M43L (primers M43L-F and M43L-R), M253L (primers M253L-F and M253L-R) and M253F (primers M253F-F and M253F-R) variants, partially overlapping primers and Q5 high-fidelity DNA polymerase (New England Biolabs) were used in a 2-stage PCR. To construct M77L variant (primers M77L-F and M77L-R), DNA polymerase used was substituted with PfuUltra high-fidelity DNA polymerase AD (Agilent Technologies).

Saturation mutagenesis was performed on positions 56, 109, 227, 306, 312 and 374 of DyP4 using pET-24a(+)-OsmY-DyP4 variant 3F6 as template. For position 312, non-overlapping primers were used (Table 1). For positions 56, 109, 227, 306 and 374, a 4-primer method was applied using 2 flanking primers (BamHI-DyP4-Fwd and DyP4-Rev) and 2 internal primers (Table 1). Q5 high-fidelity DNA polymerase (New England Biolabs) was used in all PCRs.

### Protein expression and purification

For protein expression, plasmid was freshly transformed into *E. coli* BL21 (DE3). Cells were grown in 2× TY media supplemented with 50 μg/mL kanamycin at 37 °C. When OD_600_ reached 0.5–0.6, 1 mM isopropyl β-d-1-thiogalactopyranoside (IPTG) was added to induce protein expression and temperature was lowered to 25 °C. After 24 h, cells were harvested and pellets were stored at − 20 °C.

For protein purification, cells were resuspended in pre-chilled buffer A (50 mM Tris–HCl pH 8.5, 1 mM EDTA) supplemented with DNase, RNase, protease inhibitor and lysozyme, and were lysed by sonication (Vibra-Cell ultrasonic liquid processors; Sonics & Materials; Newtown, USA). Lysed cells were centrifuged and supernatant was loaded onto a 5-mL HiTrap Q HP anion exchange chromatography column (GE Healthcare Life Sciences; Pittsburgh, USA) pre-equilibrated with buffer A. After washing with 5 column volumes (CVs) of buffer A, protein was eluted with a linear gradient of NaCl (0–1 M) in buffer A. Protein was subsequently diluted 10× with buffer B (25 mM acetate pH 4.0) to adjust the NaCl concentration, before loading onto a 5-mL HiTrap SP HP cation exchange chromatography column (GE Healthcare Life Sciences) pre-equilibrated with buffer B. After washing with 5 CVs of buffer B, protein was eluted with a linear gradient of NaCl (0–1 M) in buffer B. Fractions containing target protein were pooled and loaded onto HiLoad 26/600 Superdex 75 pg column (GE Healthcare Life Sciences) pre-equilibrated with buffer C [0.1 M citrate–0.2 M Na_2_HPO_4_ pH 4.0, 100 mM NaCl, 10% (v/v) glycerol]. Purified DyP4 protein was immediately flash cooled in liquid nitrogen and stored in − 80 °C.

### UV–visible spectroscopy and concentration measurement of purified DyP4 WT and variants

Purified DyP4 stored at − 80 °C was thawed and transferred to a quartz cuvette. UV–visible spectra were collected using a UV-3100PC spectrophotometer (VWR; Lutterworth, UK). Spectra between 250–800 nm were recorded at ambient temperature for all proteins in buffer C, typically at concentrations of 3–6 μM. DyP4 protein concentration was calculated based on absorbance at 280 nm, measured using the BioPhotometer Plus (Eppendorf; Stevenage, UK), and an extinction coefficient of 29,575 M^−1^ cm^−1^ (calculated using ProtParam).

### Kinetic constants of DyP4 WT and variants

The kinetic constants of DyP4 were determined for ABTS oxidation. All ABTS stock solutions were prepared in 0.1 M citric acid–0.2 M Na_2_HPO_4_ buffer pH 3.4 and 1 mM hydrogen peroxide was prepared in deionised water. The assay was performed at ambient temperature in flat-bottom clear 96-well polystyrene microplates (Greiner Bio-One; 655161). 140 μL of ABTS stock solution (final concentrations of 0.1–7.0 mM) was transferred to the microtitre plate before 10 μL of purified DyP4 (final concentrations of 0.1–0.2 μM) was added. The reaction was initiated by 50 μL of 1 mM H_2_O_2_ and formation of ABTS cation radical was recorded at 405 nm [*ε*_405_ = 36.8 mM^−1^ cm^−1^, (Otieno et al. 2016)] with Multiskan FC microplate photometer (Thermo Fisher Scientific). All experiments were performed in triplicates. The Michaelis constants (*K*_m_ and *V*_max_) were obtained by non-linear regression analysis to the Michaelis–Menten model using GraphPad Prism (GraphPad Software; San Diego, USA). The catalytic rate (*k*_cat_) was calculated using *k*_cat_= *V*_max_/(DyP4 concentration).

### Hydrogen peroxide tolerance of DyP4 and variants

The hydrogen peroxide tolerance for DyP4 was determined using ABTS oxidation. Assay procedure is similar to the determination of DyP4 kinetic constants, except 7 mM ABTS in 0.1 M citric acid–0.2 M Na_2_HPO_4_ buffer pH 3.4 and 0.15–50.00 mM H_2_O_2_ were used. All reaction rates were normalized against the reaction rate at 0.25 mM H_2_O_2_ for the respective DyP4 wildtype or variants. All experiments were performed in triplicates. The residual activity was plotted against H_2_O_2_ concentration and non-linear regression analysis to the “[inhibitor] vs. response—variable slope (four parameter)” model was performed using GraphPad Prism (GraphPad Software) to obtain IC_50_ values of H_2_O_2_.

### Acetone precipitation of protein

One volume of spent medium (350 μL) was mixed with 4 volumes of acetone (1400 μL) that was pre-chilled at − 20 °C. The mixture was vortexed thoroughly and incubated at − 80 °C for 15 min, before overnight incubation at − 20 °C. Subsequently, the mixture was centrifuged at 21,000 *g* and 4 °C for 15 min. After decanting the supernatant, residual acetone was allowed to evaporate at room temperature for 30 min. Protein pellet was dissolved in 35 μL of 1× SDS sample loading buffer and 20 μL of protein sample was analysed by SDS-PAGE.

### Proteomic analysis

Proteins present in SEC fractions 2 and 7 were first resolved by SDS-PAGE. Gel bands were excised from the SDS-PAGE gel manually, post visualization by Coomassie blue stain. The gel bands were then de-stained and proteolytically digested with trypsin to generate peptides for mass spectrometry analysis (Shevchenko et al. 2006) using liquid chromatography LC–MS/MS. Mascot software search engine was employed to process the mass spectrometry data to identify proteins from the peptide sequence reference database for *Escherichia coli* (strain B/BL21-DE3) (UniProt Proteome ID: UP000002032; 4156 entries; downloaded on 07/04/2019). The sequence of the recombinant OsmY-DyP4 protein was included as an additional entry to this reference proteome.

## Results and discussion

### Methionine substitution did not improve H_2_O_2_ tolerance of DyP4

Peroxidases use H_2_O_2_ as the electron acceptor to catalyse numerous oxidative reactions. For DyP4, the optimum H_2_O_2_ concentration for ABTS oxidation was determined to be 0.25 mM. This was consistent with a previous study that used exactly the same H_2_O_2_ concentration for the oxidation of ABTS, Mn^2+^, Reactive Blue 19 (RB19), Reactive Black 5 (RB5) and 2,6-dimethoxyphenol (DMP) (Fernandez-Fueyo et al. 2015). Other DyPs operate optimally in similar H_2_O_2_ concentration range. For example, 0.2 mM H_2_O_2_ was used for ABTS, guaiacol and DMP oxidation by bacterial DyP from *Pseudomonas putida* MET94 (PpDyP) (Brissos et al. 2017). DyP from cyanobacterium *Anabaena* sp. strain PCC 7120 (AnaPX) functioned optimally at 0.4 mM H_2_O_2_ for RB5 oxidation (Ogola et al. 2009). For DyP from *Irpex lacteus*, the optimum H_2_O_2_ concentration for ABTS oxidation was between 0.4 and 0.8 mM (Salvachua et al. 2013).

As with other DyPs, DyP4 is inhibited by H_2_O_2_. In an attempt to enhance the H_2_O_2_ tolerance of DyP4, we created 4 methionine-substituted variants (M43L M77L M253L and M253F). Our decision was guided by two reasons: (1) The amino acids methionine, cysteine, histidine, tryptophan and tyrosine are typical targets for oxidation within proteins due to the high reactivity of sulphur atoms and aromatic rings towards various reactive oxygen species (Li et al. 1995), and (2) Ogola et al. successfully enhanced the H_2_O_2_ stability of AnaPX by 2.4- to 8.2-folds through substituting methionine residues with high redox residues such as isoleucine, leucine and phenylalanine (Ogola et al. 2010).

Although all 4 methionine-substituted variants were properly folded judging by the absorption spectra of the purified proteins and were catalytically active, there was no improvement in their H_2_O_2_ tolerance (data not shown). This result prompted us to apply directed evolution to enhance H_2_O_2_ tolerance.

### N-terminal OsmY fusion resulted in extracellular DyP4 secretion

In directed evolution using *E. coli* as protein expression host, protein variants are often expressed in 96-well format. Prior to enzymatic assays, cells are lysed to release recombinant proteins using physical (e.g. freeze–thaw cycle), chemical (e.g. polymyxin B, Triton X-100, Tween 20, NP40, CHAPS and cholate etc.), enzymatic (e.g. lysozyme) or combinations of these approaches. In engineering PpDyP using directed evolution, for instance, *E. coli* cells were disrupted through multiple steps to release DyP enzymes: (i) cell harvest by centrifugation, (ii) physical-enzymatic treatment (freeze at − 80 °C for 15 min, thaw at 30 °C for 5 min, re-suspension in buffer containing lysozyme) and (iii) cell debris removal by centrifugation (Brissos et al. 2017).

To streamline the DyP4 directed evolution workflow, we fused *E. coli* OsmY to the N-terminus of DyP4 and investigated protein secretion using 2 expression hosts [*E. coli* BL21 (DE3) and *E. coli* C41 (DE3)] and 2 protein expression temperatures (30 °C and 37 °C) in 2× TY-based AIM. Using ABTS assay, we confirmed extracellular OsmY-DyP4 secretion and *E. coli* BL21 (DE3) was superior to *E. coli* C41 (DE3) based on the activity of secreted protein. In addition, an expression temperature of 30 °C resulted in higher protein secretion. By using OsmY as secretory carrier and fusion protein expression in AIM, we bypassed both (i) protein induction (i.e. addition of IPTG inducer) and (ii) cell disruption, which allows us to develop a far more elegant HTS as depicted in Additional file 1: Figure S3. Worthy of a mention, in addition to DyP4, OsmY was also a good carrier protein for β-glucosidase (53 kDa) and lipase (20 kDa) (unpublished data).

### Accelerated HTS with BENNY

**Fig. 1:**
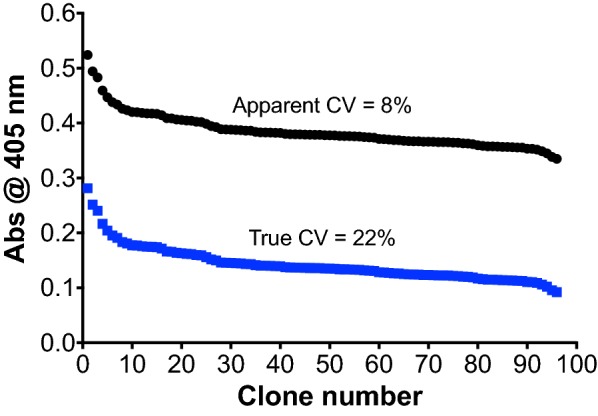
Activity values (Abs_405_) of OsmY-DyP4-catalysed ABTS oxidation in a 96-well plate, reported in a descending order (black—before background subtraction, blue—after background subtraction). The assay was conducted using a protocol streamlined with OsmY-based BENNY. The apparent coefficient of variance (CV) was calculated without subtracting the assay background, while the true CV was obtained after background subtraction

After confirming the extracellular secretion of OsmY-DyP4 using BENNY, we adapted this to 96-well format (Additional file 1: Figure S3). Based on ABTS assay, we obtained an apparent coefficient of variance (CV) of 8% for OsmY-DyP4 (Fig. 1 and Additional file 1: Figure S4). Concurrently, we cultivated *E. coli* BL21 (DE3) carrying no plasmid in a 96-well plate (denoted as background plate) to calculate the background absorbance. After subtracting the background absorbance (Abs_405_ = 0.2427), we obtained a true CV of 22%. The increase in Abs_405_ of 0.1425, on average, was therefore due solely to the enzymatic oxidation of ABTS by the secreted OsmY-DyP4.

A closer look at both the assay plate and the background plate revealed that the deviation was mainly caused by liquid evaporation from bordering wells, which resulted in higher secreted protein concentration in these wells (Additional file 1: Figure S4). If all bordering wells were excluded, the apparent and true CVs were reduced to 4% and 11%, respectively.

Therefore, BENNY-assisted ABTS assay was well suited for directed evolution to differentiate and identify improved protein variants. Worthy of note, in order to isolate OsmY-DyP4 variants of enhanced H_2_O_2_ tolerance, we intentionally increased the H_2_O_2_ concentration in our assay from 0.25 to 4 mM, representing a 16× increase. At 4 mM H_2_O_2_, DyP4 WT showed a relative activity of ~ 30%, compared to that at 0.25 mM H_2_O_2_.

### Random mutagenesis by epPCR

**Fig. 2:**
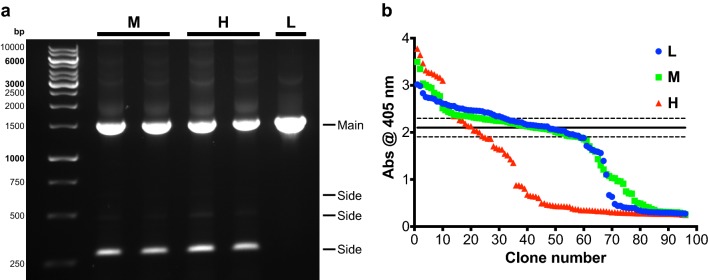
**a** Products and side products of epPCRs with high (H), medium (M) and low (L) mutation rates. **b** ABTS oxidation activity values (Abs_405_) of OsmY-DyP4 mutant libraries from the 3rd round of epPCR [blue—epPCR library with low (L) mutation rate, green—epPCR library with medium (M) mutation rate, red—epPCR library with high (H) mutation rate]. Solid and dotted horizontal lines represent the average activity of parental strain (i.e. 2A5) and one standard deviation (SD) above/below the average value, respectively

As this was our first directed evolution attempt on DyP4, we created random mutagenesis libraries of OsmY-DyP4 using 3 epPCR conditions (H, M and L). Important to note, mutations were only targeted to the sequence encoding for DyP4, leaving the OsmY sequence unaltered. The error rate of Taq DNA polymerase was manipulated by increasing Mg^2+^ concentration, adding Mn^2+^, applying imbalanced nucleotide concentration or utilizing combinations of these factors (Wong et al. 2006). As evidenced in Fig. 2a, at high and medium error rates, we observed a reduction in the PCR product yield and appearance of multiple side products. Therefore, combining increased Mg^2+^ concentration and Mn^2+^ addition (condition H) gave the highest error rate. A combination of increased Mg^2+^ concentration and imbalanced nucleotide concentration (condition M) gave the medium error rate. Finally, adding 0.01 mM Mn^2+^ alone (condition L) gave the lowest error rate.

This was further confirmed by screening these epPCR libraries using BENNY-assisted ABTS assay (Fig. 2b). Most of the clones (77.42%; Additional file 1: Figure S5) in the library prepared with condition H were either inactive or not as active as the parental strains (wells B2, E6 and G11). These percentages were much lower in the libraries prepared with conditions M (41.94%) and L (40.86%).

### Improved OsmY-DyP4 variants isolated from all epPCR conditions (H, M and L)

In total, we performed 3 rounds of random mutagenesis using epPCR. In the 1st and 2nd rounds, we screened 558 clones [2 96-well plates from each epPCR condition (H, M, L)]. In the 3rd round, 1116 clones [4 96-well plates from each epPCR condition (H, M, L)] were screened. The best variant from each epPCR round (OsmY-1D2 from 1st round and OsmY-2A5 from 2nd round) was used as parental template for the subsequent epPCR round. In the 4th round, we performed saturation mutagenesis on each mutated amino acid position identified (56, 109, 227, 306, 312 and 374), using OsmY-3F6 as template DNA.

As summarized in Table 2, OsmY-DyP4 variants with enhanced total activity under screening conditions were identified from all epPCR conditions (H, M, L). DNA sequencing of this set of improved variants showed that nucleotide substitutions were predominantly AT→GC transitions (63.6%; Additional file 1: Table S1), consistent with the expected mutational spectrum of epPCR using Taq DNA polymerase (Wong et al. 2006; Tee and Wong 2013). On average, there were 1.82 nucleotide substitutions per DyP4 gene, which was deemed appropriate for directed evolution.

**Table 2:** A list of OsmY-DyP4 variants and the mutations in DyP4-coding sequences verified by DNA sequencing

OsmY-DyP4 and variants	Mutagenic rate of epPCR library	Missense mutations	Silent mutations
OsmY-WT*	N/A	N/A	N/A
OsmY-1D2*	L	*N312S (A→G)*	*D241 (T→C)* *I444 (C→T)*
OsmY-1D7	H	*A306V (C→T)*	*R323 (T→C)*
OsmY-2A5*	M	*I56V (A→G)* N312S (A**→**G)	D241 (T→C) I444 (C→T) *G73* *(T→A)* *L245 (G→T)*
OsmY-2C2	M	*H374R (A→G)* N312S (A**→**G)	D241 (T**→**C) I444 (C**→**T)
OsmY-2F8	H	*N227D (A→G)* N312S (A**→**G)	D241 (T**→**C) I444 (C**→**T)
OsmY-3F6*	L	*K109R (A→G)* N312S (A**→**G) I56V (A**→**G)	D241 (T**→**C) I444 (C**→**T) G73 (T**→**A) L245 (G**→**T)
OsmY-4D4	N/A	*N227S* N312S (A**→**G) I56V (A**→**G) K109R (A**→**G)	D241 (T**→**C) I444 (C**→**T) G73 (T**→**A) L245 (G**→**T)

Clones labelled with an asterisk * were used as parental template for the subsequent round of mutagenesis; mutations in italics represent nucleotide substitutions added to a parental template

**Fig. 3:**
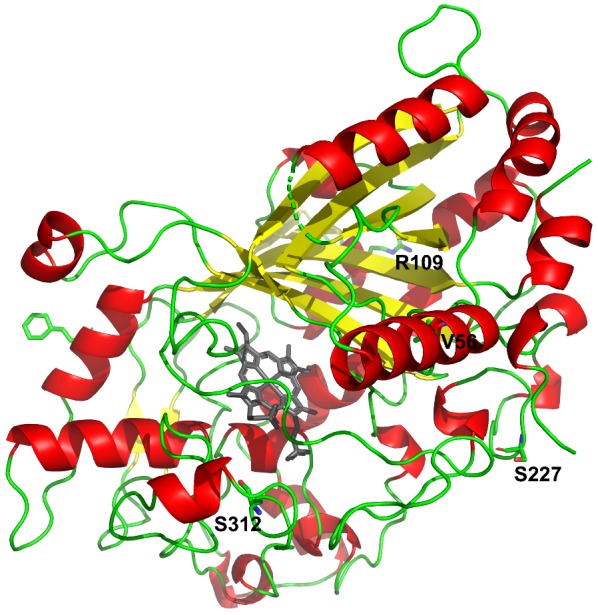
Protein model of 4D4 variant, created with PyMOL using crystal structure of DyP4 F194Y variant (PDB 6FSK) as template. Missense mutations in 4D4 are indicated (I56V, K109R, N227S and N312S)

The best variant, OsmY-4D4, carries 4 amino acid substitutions (I56V, K109R, N227S and N312S; Fig. 3 and Additional file 1: Figure S6) along with 4 other silent mutations (Table 2). The crystal structures of F194Y (PDB 6FSK) and F194W (PDB 6FSL) variants of DyP4 were recently released. Residues 56 and 109 are located within α-helices, while the other two residues (227 and 312) are located in turns. None of these residues is in the vicinity of haem.

To further investigate the amino acid substitutions found in directed evolution, we looked at 3 aspects more closely solvent accessibility, B-factor and H-bond formation. Of the 4 residues, residue 109 is the only surface-exposed residue. The average B-factor of residue 109 (21.5678) is also slightly higher than protein average (21.146) and those of residues 56 (17.6775), 227 (15.9750) and 312 (15.4975). Interestingly, K109R and N312S substitutions resulted in higher number of H-bonds formed (3 H-bonds in R109 vs. 1 H-bond in K109 and 4 H-bonds in S312 vs. 3 H-bonds in N312).

### DyP4 variants showed higher protein yield, specific activity and H_2_O_2_ tolerance

The enhanced total activity of OsmY-DyP4 variants in Table 2 could be due to one or more of the following factors: (1) increased protein expression or yield, (2) enhanced H_2_O_2_ tolerance and (3) increased extracellular protein secretion. To determine the effects of mutations found, we removed the N-terminal OsmY of WT, 3F6 and 4D4, expressed and purified these 3 proteins, and performed further characterizations.

**Fig. 4:**
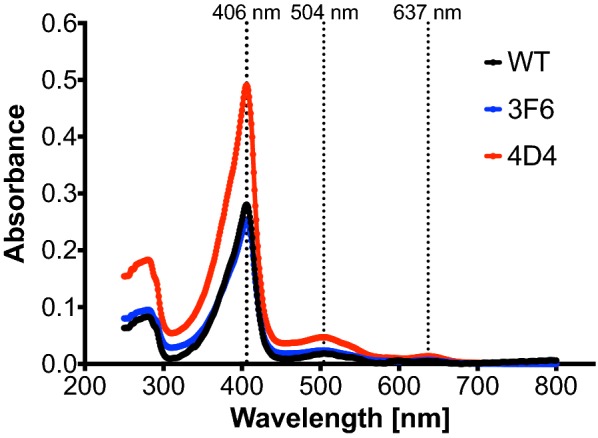
UV-vis spectra of purified WT (black), 3F6 (blue) and 4D4 (red), indicating the presence of a Soret band at 406 nm with a slight shoulder at ~ 385 nm, Q band at 504 nm and a charge-transfer (CT) band at 637 nm

Judging on the reddish colour of the cell pellets (Additional file 1: Figure S7) and the protein content of cell extract (Additional file 1: Figure S8), the protein yield of 3F6 and 4D4 was higher than that of WT. From 200-mL shake flask cultures, the estimated amounts of purified protein obtained were 1.16 mg for WT, 1.20 mg for 3F6 and 2.70 mg for 4D4. This could potentially explain marginally higher protein secretion that we observed for OsmY-3F6 and OsmY-4D4 when we loaded the acetone-precipitated protein samples onto SDS-PAGE (Additional file 1: Figure S9). After protein purification, all 3 purified proteins exhibited a distinctive Soret band at 406 nm with a slight shoulder at ~ 385 nm (Fig. 4). Q band at 504 nm and a charge-transfer (CT) band at 637 nm were also clearly visible.

Using the purified proteins, we investigated the kinetic parameters for ABTS oxidation (Table 3). The *k*_cat_ values of 3F6 and 4D4 were found to be 5.02 s^−1^ and 4.52 s^−1^, respectively. These are slightly higher than WT’s *k*_cat_ of 3.86 s^−1^. Variants 3F6 and 4D4 showed lower *K*_m_ values of 0.427 mM and 0.390 mM, respectively, compared to WT (0.895 mM). This was unexpected as we used a relatively high ABTS concentration in our HTS, 6.8 mM, which is 7.6× above the WT’s *K*_m_ value. In fact, the WT’s *K*_m_ value we determined (0.895 mM) was almost identical to a previously reported value (0.779 mM) (Fernandez-Fueyo et al. 2015). The combination of higher *k*_cat_ and lower *K*_m_ resulted in a 2.7-fold improvement in specific activity for both 3F6 and 4D4.

**Table 3:** Kinetic parameters for ABTS oxidation of DyP4 and its variants

DyP4 and variants	*K*_m_ (mM)	*V*_max_ (mM^−1^ s^−1^)	*k*_cat_ (s^−1^)	*k*_cat_/*K*_m_ (mM^−1^ s^−1^)
WT	0.895 ± 0.065	6.779 ± 0.142 × 10^−4^	3.86 ± 0.08	4.31 ± 0.41
3F6	0.427 ± 0.055	6.877 ± 0.202 × 10^−4^	5.02 ± 0.15	11.78 ± 1.86
4D4	0.390 ± 0.059	6.966 ± 0.233 × 10^−4^	4.52 ± 0.15	11.60 ± 2.14

**Fig. 5:**
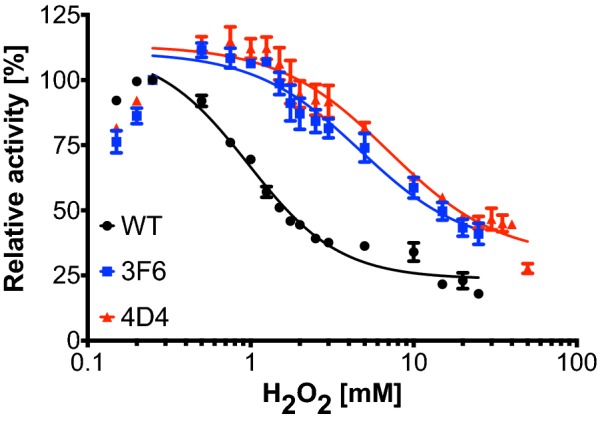
Relative ABTS oxidation activities of purified WT (black), 3F6 (blue) and 4D4 (red), at increasing H_2_O_2_ concentrations from 0.15 to 50.00 mM, with ABTS concentration kept at 7 mM. The WT activity at 0.25 mM H_2_O_2_was arbitrarily set as 100% and used as a reference point

We also conducted ABTS assay in the presence of increasing H_2_O_2_ concentration (0.15–50.00 mM), with ABTS concentration kept at 7 mM. In Fig. 5, the activities at 0.25 mM H_2_O_2_ (the most optimal H_2_O_2_ concentration for ABTS oxidation) for WT and variants were arbitrarily set as 100% and used as reference points. Activities of WT, 3F6 and 4D4 at all other H_2_O_2_ concentrations were benchmarked against their respective reference. It became immediately apparent that the curves corresponding to 3F6 and 4D4 were shifted upwards and rightwards, indicating higher activity, a shift to higher optimal H_2_O_2_ concentration and higher H_2_O_2_ tolerance. Upon fitting the curves to an inhibitory dose–response curve with a variable slope (4 parameters), the IC_50_ value was increased from 0.97 mM (WT) to 4.67 mM (3F6) and 7.03 mM (4D4), as summarized in Table 4. In other words, the H_2_O_2_ tolerance of 4D4 was improved 7×, comparing its IC_50_ value to that of WT.

**Table 4:** H_2_O_2_ tolerance data of DyP4 and its variants were fitted to an inhibitory dose–response curve with a variable slope (4 parameters), using GraphPad software

Best fit values	WT	3F6	4D4
Bottom	23.7 ± 1.6	35.4 ± 8.9	32.0 ± 6.8
Top	111.7 ± 5.9	110.8 ± 4.4	113.3 ± 3.8
HillSlope	1.6 ± 0.2	1.3 ± 0.4	1.3 ± 0.3
IC_50_	0.97 ± 0.10	4.67 ± 1.06	7.03 ± 1.33
*R* ^2^	0.9680	0.9131	0.9315

### Protein production rate is likely faster than protein secretion rate

When we compared the cell pellets of DyP4 and variants with or without N-terminal OsmY fusion partner (Additional file 1: Figure S7), we noticed apparent colour difference between the two sets. The set with OsmY displayed much lighter reddish colour, which was expected for a system that allows extracellular protein secretion. Consistent with the set without OsmY, OsmY-3F6 and OsmY-4D4 showed slightly darker reddish colour compared to OsmY-WT, again suggesting higher protein yield of these two variants. This result also indicated that a portion of OsmY-DyP4 was not secreted. To understand the reason behind, we purified the OsmY-DyP4 from the cell pellets by following the identical protein purification scheme as DyP4.

**Fig. 6:**
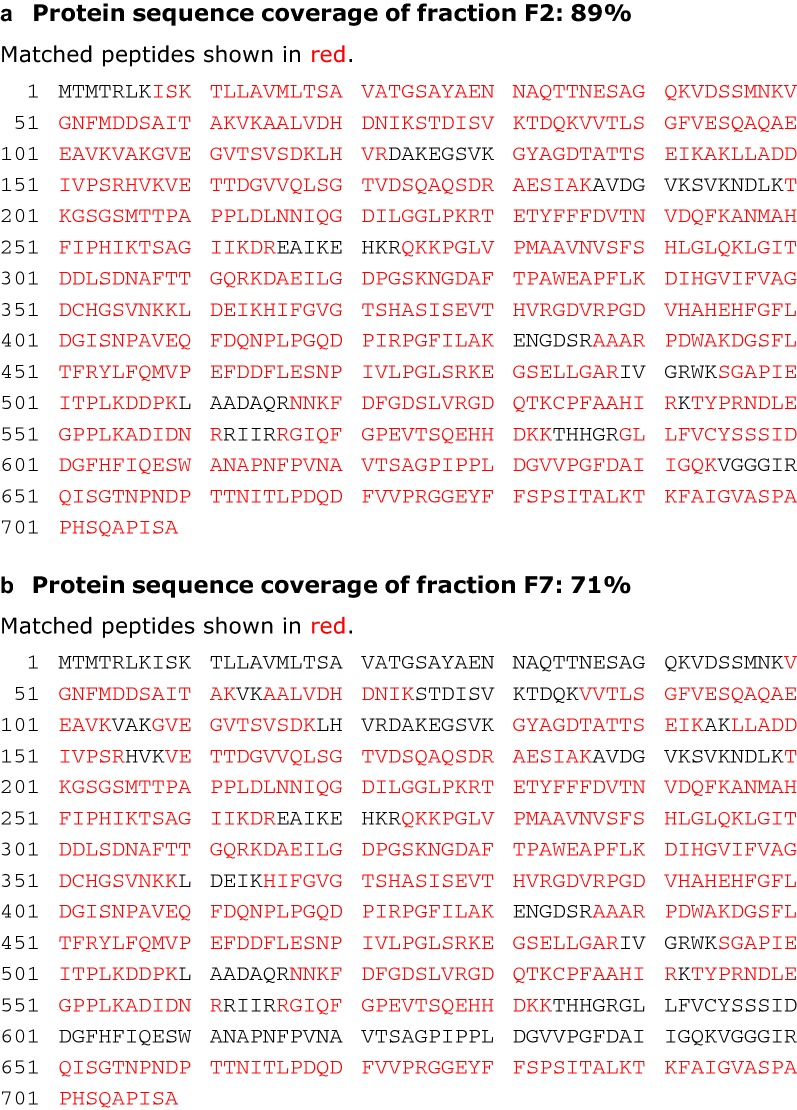
Mass spectrometry data of SEC fractions F2 (**a**) and F7 (**b**) proteins. Mascot database searching against the *Escherichia coli* (strain B/BL21-DE3) reference proteome, plus the recombinant OsmY-DyP4 sequence, led to matching to this recombinant protein. Matched peptides are shown in red against the full-length amino acid sequence of the recombinant protein

In the final size exclusion chromatographic (SEC) step, we noticed that the haem-containing protein peak had a slight right shoulder (Additional file 1: Figure S10), indicating the presence of a slightly smaller protein. This was further confirmed when we loaded all collected protein fractions (F2 to F7) onto SDS-PAGE, where we saw two distinct protein bands (Additional file 1: Figure S10). We excised the protein band in F2 (larger *M*_w_) and the protein band in F7 (smaller *M*_w_), and conducted proteomic analysis. Mass spectrometry data acquired by LC–MS/MS led to both F2 and F7 matching to the recombinant OsmY-DyP4, with a lower sequence coverage for F7 (71%) relative to F2 (89%). Overlaying identified peptides to the amino acid sequence of OsmY-DyP4 fusion protein (Fig. 6) suggests a potential N-terminal truncation of the F2 protein to generate the F7 protein. The data suggested that the truncation likely occurred within the first 50 amino acids of OsmY. In other words, the signal peptide of OsmY (positions 1–28) was proteolytically cleaved, resulting in no secretion of this truncated protein pool. This signal peptide of OsmY was previously shown to play a key role in protein secretion (Qian et al. 2008).

Taken together, our experimental data suggested that (1) protein production rate of OsmY-DyP4 is likely faster than its extracellular secretion rate, and (2) the N-terminal region of OsmY, which encompasses its first 28 amino acids, is susceptible to proteolytic cleavage. These two factors potentially contribute to retention of small amount of DyP4 within the cell.

## Conclusion

In conclusion, we were able to apply *E. coli* OsmY in a BENNY-assisted HTS system to significantly streamline the workflow of directed DyP4 evolution. Using this simplified scheme, we successfully isolated DyP4 variants that show multiple desirable phenotypes including higher protein yield, higher specific activity and higher H_2_O_2_ tolerance. We are now further engineering 4D4 variant for the oxidation of S-type phenolic lignin units (Camarero et al. 1994; Pardo et al. 2013) such as syringaldehyde, acetosyringone and sinapic acid etc. (on-going work).

The application of OsmY-based BENNY can further be extended to engineering other enzyme types, expressing toxic proteins and simplifying downstream processing in recombinant enzyme production. We are currently expanding the BENNY toolbox by (1) identifying new secretory proteins, (2) engineering OsmY and other related proteins for higher secretory phenotypes and (3) extending the application of OsmY to other bacterial hosts, encompassing both Gram-positive and Gram-negative bacteria (on-going work). Concurrently, we see exciting development in the field of BENNY reported by other research groups, such as identification of useful secretory carriers [e.g. YebF (Zhang et al. 2006) and Hly (Ruano-Gallego et al. 2019)] and engineering *E. coli* cells showing hyper secretory phenotypes (e.g. *E. coli* BW25113 *ΔyaiW* and *E. coli* BW25113 *ΔgfcC*) (Natarajan et al. 2017).

## Additional file

**Additional file 1.** Supplementary material.

## Data Availability

Not applicable.

## Abbreviations

Abs_405_: absorbance at 405 nm
ABTS: 2,2′-azino-bis(3-ethylbenzothiazoline-6-sulphonic acid)
AIM: auto induction medium
AnaPX: dye-decolorizing peroxidase from cyanobacterium *Anabaena* sp. strain PCC 7120
BENNY: bacterial extracellular protein secretion system
CV: coefficient of variance
CVs: column volumes
dATP: deoxyadenosine triphosphate
dCTP: deoxycytidine triphosphate
dGTP: deoxyguanosine triphosphate
DMP: 2,6-dimethoxyphenol
dNTP: deoxynucleotide
dTTP: deoxythymidine triphosphate
DyP: dye-decolorizing peroxidase
DyP4: dye-decolorizing peroxidase 4 from *Pleurotus ostreatus* strain PC15
*E. coli*: *Escherichia coli*
epCPR: error-prone polymerase chain reaction
HTS: high-throughput screening
IC_50_: half maximal inhibitory concentration
IPTG: isopropyl β-d-1-thiogalactopyranoside
LiP: lignin peroxidase
MnP: manganese peroxidase
*M*_w_: molecular weight
OD_600_: optical density at 600 nm
OsmY: osmotically-inducible protein Y
PCR: polymerase chain reaction
*P. ostreatus*: *Pleurotus ostreatus*
PpDyP: dye-decolorizing peroxidase from *Pseudomonas putida* MET94
RB5: Reactive Black 5
RB19: Reactive Blue 19
SDS-PAGE: sodium dodecyl sulphate polyacrylamide gel electrophoresis
SEC: size exclusion chromatography
WT: wildtype
