# Supplementary material for: Accelerated directed evolution of dye-decolorizing peroxidase using a bacterial extracellular protein secretion system (BENNY)
Source: Bioresour Bioprocess. 2019 May 31;6(1):20. doi: 10.1186/s40643-019-0255-7 (PMC6544594; doi:10.1186/s40643-019-0255-7)

Additional Information

Accelerated directed evolution of dye-decolorizing peroxidase using a bacterial extracellular protein secretion system (BENNY)

Abdulrahman HA Alessa[1,+], Kang Lan Tee[1,+,‡], David Gonzalez-Perez[1], Hossam EM Omar Ali[1], Caroline A Evans[1], Alex Trevaskis[1], Jian-He Xu[2], Tuck Seng Wong[1,‡]

[1]Department of Chemical & Biological Engineering and Advanced Biomanufacturing Centre, University of Sheffield, Sir Robert Hadfield Building, Mappin Street, Sheffield S1 3JD, United Kingdom; [2]Laboratory of Biocatalysis and Bioprocessing, State Key Laboratory of Bioreactor Engineering, East China University of Science and Technology, 130 Meilong Road, Shanghai 200237, PR China.

[+]Both authors contributed equally.

[‡]Address correspondence to:

Dr. Kang Lan Tee
Email: k.tee@sheffield.ac.uk
Tel: +44 (0)114 222 7591
Fax: +44 (0)114 222 7501

or

Dr. Tuck Seng Wong
Email: t.wong@sheffield.ac.uk
Tel: +44 (0)114 222 7591
Fax: +44 (0)114 222 7501

TABLES

Table S1: Nucleotide substitution pattern of variants identified from epPCR libraries.

**Type**	**Transition**		**Transversion**			
	**A→G | T→C**	**G→A | C→T**	**A→C | T→G**	**A→T | T→A**	**G→C | C→G**	**G→T | C→A**
**Number**	7	2	0	1	0	1
**%**	63.6%	18.2%	0.0%	9.1%	0.0%	9.1%
**Subtotal**	81.8%		18.2%			

FIGURES

Figure S1: DNA sequence encoding both *Escherichia coli* osmotically-inducible protein Y (OsmY; highlighted in cyan) and dye-decolorizing peroxidase 4 from *Pleurotus ostreatus* strain PC15 (DyP4; highlighted in green). A linker encoding GSGS (highlighted in magenta) was inserted between both gene sequences. The sequence was codon-optimized for recombinant expression in *E. coli*.

Sequence: OsmY-DyP4.dna (Linear / 2130 bp)
Features: 3 visible, 3 total

```
atgaccatgacccgtctgaagattagcaaaaccctgctggcggtg   45
atgctgaccagcgcggtggcgaccggtagcgcgtatgcggagaac   90
aacgcgcagaccaccaacgaaagcgcgggccaaaaggttgacagc   135
agcatgaacaaagtgggtaacttcatggacgatagcgcgattacc   180
gcgaaggttaaagcggcgctggttgaccacgataacattaagagc   225
accgacatcagcgttaagaccgatcagaaagtggttaccctgagc   270
ggctttgtggaaagccaggcgcaagcggaggaagcggtgaaggtt   315
gcgaaaggtgtggagggcgttaccagcgtgagcgacaaactgcac   360
gttcgtgatgcgaaggaaggtagcgtgaaaggttacgcgggcgat   405
accgcgaccaccagcgagattaaggcgaaactgctggcggacgat   450
atcgttccgagccgtcacgtgaaggttgaaaccaccgacggcgtg   495
gttcaactgagcggtaccgtggacagccaggcgcaaagcgatcgt   540
gcggagagcatcgcgaaagcggttgacggcgtgaagagcgttaaa   585
aacgatctgaagaccaaaggtagcggatccATGACCACCCCGGCG   630
CCGCCGCTGGACCTGAACAACATCCAGGGTGATATTCTGGGTGGC   675
CTGCCGAAGCGTACCGAGACCTACTTCTTTTTCGACGTGACCAAC   720
GTTGATCAATTTAAAGCGAACATGGCGCACTTCATCCCGCACATT   765
AAGACCAGCGCGGGCATCATTAAAGACCGTGAGGCGATCAAGGAA   810
CACAAACGTCAGAAGAAACCGGGTCTGGTGCCGATGGCGGCGGTG   855
AACGTTAGCTTTAGCCACCTGGGCCTGCAGAAGCTGGGTATCACC   900
GACGATCTGAGCGATAACGCGTTCACCACCGGCCAGCGTAAGGAC   945
GCGGAGATTCTGGGCGATCCGGGTAGCAAAAACGGTGATGCGTTT   990
ACCCCGGCGTGGGAAGCGCCGTTCCTGAAGGACATCCACGGTGTG   1035
ATTTTTGTTGCGGGCGATTGCCACGGTAGCGTGAACAAGAAACTG   1080
GACGAGATCAAACACATTTTCGGCGTTGGTACCAGCCACGCGAGC   1125
ATCAGCGAAGTGACCCATGTTCGTGGCGACGTGCGTCCGGGTGAT   1170
GTTCACGCGCACGAGCACTTTGGCTTCCTGGATGGTATTAGCAAC   1215
CCGGCGGTTGAACAGTTTGATCAGAACCCGCTGCCGGGTCAGGAC   1260
CCGATCCGTCCGGGTTTCATTCTGGCGAAGGAGAACGGTGACAGC   1305
CGTGCGGCGGCGCGTCCGGACTGGGCGAAAGATGGTAGCTTTCTG   1350
ACCTTCCGTTACCTGTTCCAAATGGTGCCGGAGTTTGACGATTTC   1395
CTGGAAAGCAACCCGATCGTTCTGCCGGGCCTGAGCCGTAAAGAG   1440
GGTAGCGAACTGCTGGGTGCGCGTATTGTGGGCCGTTGGAAAAGC   1485
GGTGCGCCGATCGAGATTACCCCGCTGAAGGACGATCCGAAACTG   1530
GCGGCGGATGCGCAGCGTAACAACAAGTTTGACTTCGGCGATAGC   1575
CTGGTTCGTGGTGACCAAACCAAGTGCCCGTTCGCGGCGCACATC   1620
CGTAAAACCTACCCGCGTAACGATCTGGAAGGTCCGCCGCTGAAA   1665
GCGGACATCGATAACCGTCGTATCATTCGTCGTGGCATTCAGTTT   1710
GGTCCGGAAGTGACCAGCCAAGAACACCACGACAAGAAAACCCAC   1755
CACGGCCGTGGTCTGCTGTTCGTTTGCTATAGCAGCAGCATCGAC   1800
GATGGCTTTCACTTCATTCAGGAAAGCTGGGCGAACGCGCCGAAC   1845
TTTCCGGTGAACGCGGTTACCAGCGCGGGTCCGATCCCGCCGCTG   1890
GATGGTGTGGTTCCGGGTTTCGATGCGATCATTGGCCAGAAAGTG   1935
GGTGGCGGTATCCGTCAAATTAGCGGTACCAACCCGAACGACCCG   1980
ACCACCAACATTACCCTGCCGGACCAGGATTTTGTGGTTCCGCGT   2025
GGCGGTGAGTACTTTTTCAGCCCGAGCATCACCGCGCTGAAGACC   2070
AAATTCGCGATTGGCGTTGCGAGCCCGGCGCCGCACAGCCAAGCG   2115
CCGATCAGCGCGTAA   2130
```

Figure S2: Plasmid map of pET-24a(+)-OsmY-DyP4.

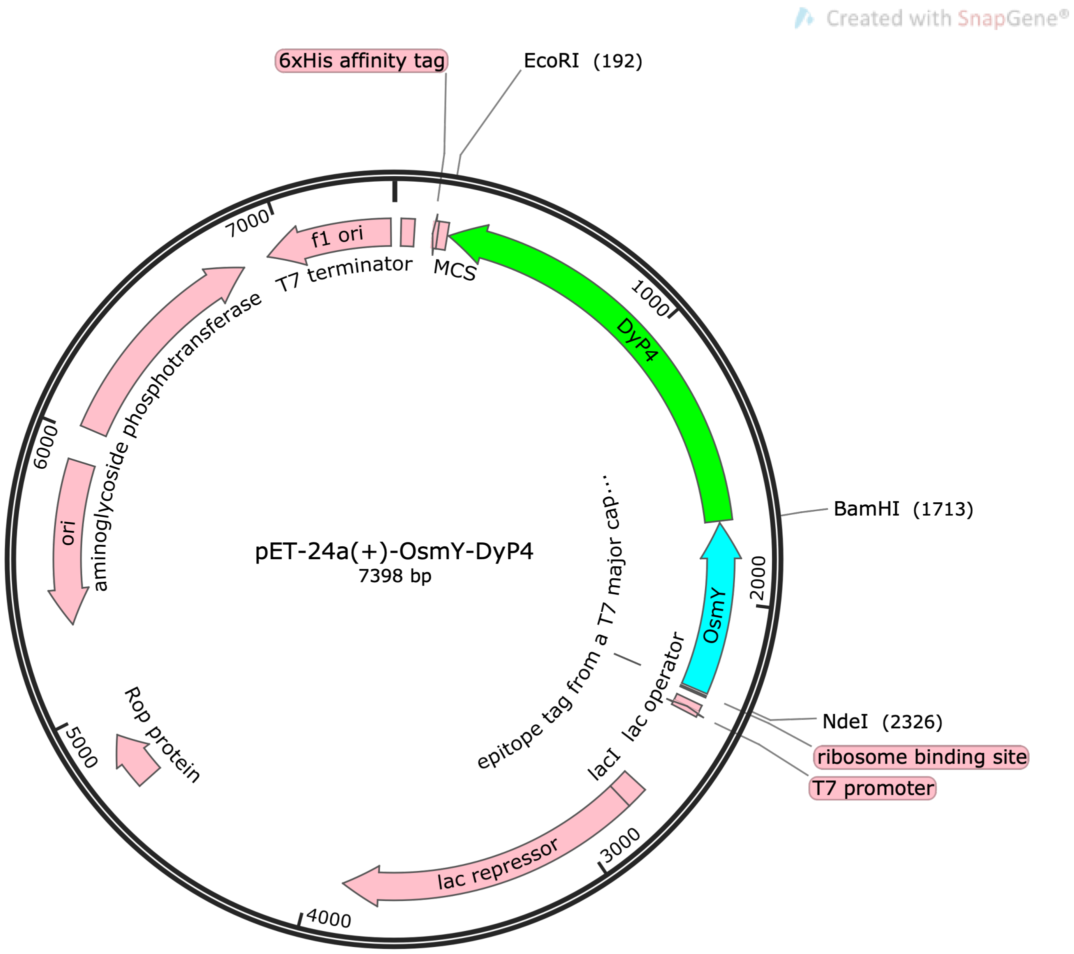

Figure S3: A streamlined BENNY-assisted high-throughput screening (HTS) used in this study.

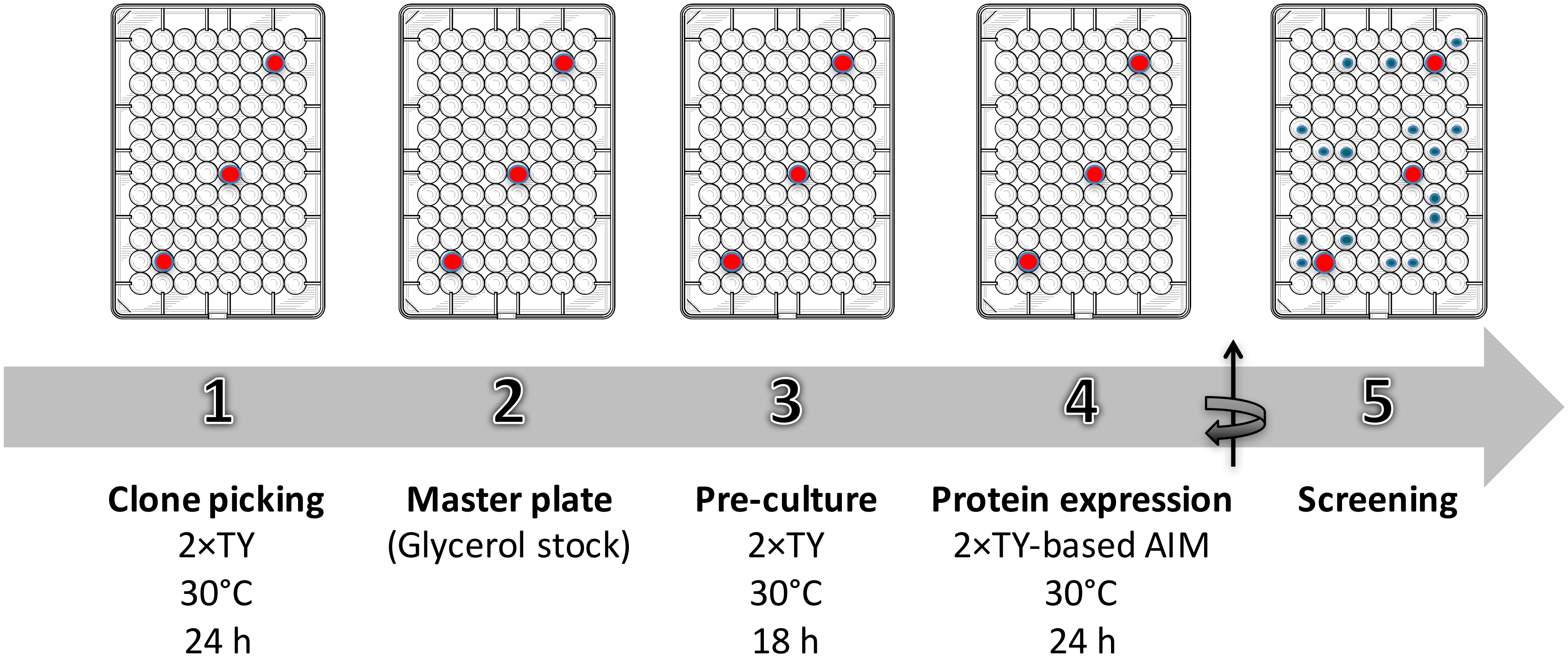

Figure S4: (**Top**) ABTS oxidation activity values (Abs_{405}) of *E. coli* BL21 (DE3) carrying no plasmid (background plate). (**Middle**) ABTS oxidation activity values (Abs_{405}) of *E. coli* BL21 (DE3) harbouring plasmid pET-24a(+)-OsmY-DyP (assay plate). (**Bottom**) Absorbance values after background subtraction. Shading intensity was directly proportional to the value. Average, standard deviation (STDEV) and coefficient of variance (CV) were calculated for the entire 96-well plate and for internal wells by excluding all bordering wells.

Background [BL21 (DE3)]

	1	2	3	4	5	6	7	8	9	10	11	12
A	0.2645	0.2579	0.2589	0.2626	0.2580	0.2461	0.2515	0.2497	0.2577	0.2554	0.2600	0.2697
B	0.2574	0.2462	0.2407	0.2435	0.2432	0.2370	0.2383	0.2419	0.2349	0.2370	0.2269	0.2549
C	0.2445	0.2401	0.2388	0.2362	0.2370	0.2358	0.2323	0.2413	0.2273	0.2357	0.2380	0.2486
D	0.2424	0.2413	0.2386	0.2359	0.2429	0.2313	0.2301	0.2423	0.2317	0.2381	0.2390	0.2425
E	0.2554	0.2436	0.2478	0.2334	0.2353	0.2354	0.2394	0.2337	0.2403	0.2309	0.2355	0.2405
F	0.2556	0.2449	0.2360	0.2341	0.2294	0.2344	0.2436	0.2309	0.2387	0.2286	0.2390	0.2410
G	0.2501	0.2431	0.2313	0.2396	0.2366	0.2303	0.2383	0.2329	0.2387	0.2279	0.2358	0.2477
H	0.2668	0.2602	0.2490	0.2522	0.2478	0.2421	0.2488	0.2437	0.2541	0.2440	0.2493	0.2565

	Whole plate
Average	0.2427

Assay plate [BL21 (DE3) harbouring pET24a-OsmY-DyP4 WT]

	1	2	3	4	5	6	7	8	9	10	11	12
A	0.4941	0.4143	0.4336	0.4043	0.3926	0.3632	0.3644	0.3880	0.3874	0.3871	0.3861	0.4197
B	0.4470	0.3887	0.3661	0.3778	0.3627	0.3658	0.3555	0.3653	0.3776	0.3642	0.3597	0.3794
C	0.4065	0.3735	0.3741	0.3764	0.3786	0.3561	0.3573	0.3585	0.3567	0.3651	0.3487	0.3668
D	0.4260	0.3788	0.3745	0.3758	0.3532	0.3449	0.3349	0.3554	0.3384	0.3517	0.3533	0.3564
E	0.4185	0.3777	0.3765	0.3762	0.3691	0.3669	0.3803	0.3711	0.3706	0.3617	0.3986	0.3826
F	0.4381	0.3787	0.3795	0.3884	0.3689	0.3828	0.3661	0.3945	0.3754	0.3699	0.3576	0.3839
G	0.4830	0.3830	0.4057	0.4089	0.3797	0.3575	0.3654	0.3861	0.3822	0.3677	0.3671	0.3824
H	0.5241	0.4593	0.4169	0.4234	0.4047	0.4202	0.4174	0.4018	0.4085	0.3790	0.4175	0.4024

	Whole plate	Removing bordering well
Average	0.3852	0.3701
STDEV	0.0319	0.0146
CV	8%	4%

Assay Plate Minus Background

	1	2	3	4	5	6	7	8	9	10	11	12
A	0.2514	0.1716	0.1909	0.1616	0.1499	0.1205	0.1217	0.1453	0.1447	0.1444	0.1434	0.1770
B	0.2043	0.1460	0.1234	0.1351	0.1200	0.1231	0.1128	0.1226	0.1349	0.1215	0.1170	0.1367
C	0.1638	0.1308	0.1314	0.1337	0.1359	0.1134	0.1146	0.1158	0.1140	0.1224	0.1060	0.1241
D	0.1833	0.1361	0.1318	0.1331	0.1105	0.1022	0.0922	0.1127	0.0957	0.1090	0.1106	0.1137
E	0.1758	0.1350	0.1338	0.1335	0.1264	0.1242	0.1376	0.1284	0.1279	0.1190	0.1559	0.1399
F	0.1954	0.1360	0.1368	0.1457	0.1262	0.1401	0.1234	0.1518	0.1327	0.1272	0.1149	0.1412
G	0.2403	0.1403	0.1630	0.1662	0.1370	0.1148	0.1227	0.1434	0.1395	0.1250	0.1244	0.1397
H	0.2814	0.2166	0.1742	0.1807	0.1620	0.1775	0.1747	0.1591	0.1658	0.1363	0.1748	0.1597

	Whole plate	Removing bordering well
Average	0.1425	0.1273
STDEV	0.0319	0.0146
CV	22%	11%

Figure S5: ABTS oxidation activity values (Abs_{405}) of three OsmY-DyP4 libraries from the 3rd round of random mutagenesis with epPCR. (**Top**) epPCR library with low (L) mutation rate. (**Middle**) epPCR library with medium (M) mutation rate. (**Bottom**) epPCR library with high (H) mutation rate. In all 3 plates, numbers in red (wells B2, E6 and G11) represent the activities of parental strain (*i.e.*, 2A5). Using these 9 values, the average activity of parental strain is determined to be 2.1020 ± 0.1966 (average ± 1 SD). Values above 2.2986 (average + 1 SD) are indicated with a green dot, values below 1.9053 (average - 1 SD) are indicated with a red dot, and values that fall within the range of 1.9053 to 2.2986 (average ± 1 SD) are indicated with an amber dot.

Low mutation rate (L)

	1	2	3	4	5	6	7	8	9	10	11	12
A	3.0168	2.7325	0.3084	2.6126	2.7281	1.5628	0.2965	2.6557	0.3031	1.6728	2.9854	0.3397
B	2.8321	2.5064	0.2835	2.4599	0.6319	2.1593	2.0365	2.3203	2.2247	2.0727	2.4478	1.3952
C	0.3220	0.4794	2.0434	2.0583	1.5869	0.3944	1.8808	2.1320	0.2936	0.2975	2.4203	2.4840
D	0.2876	2.4027	2.2735	2.2956	2.1808	0.3024	0.4016	1.9612	1.6291	1.6010	0.4093	2.5603
E	0.3518	1.1025	2.1261	2.1620	0.6677	1.9284	2.1531	2.0346	1.7136	0.2782	1.9859	2.5228
F	2.2177	2.4239	2.6022	2.2113	0.2962	2.1261	0.2728	0.4398	1.9383	1.8842	0.3201	2.5759
G	2.5048	0.3934	2.3427	2.4689	0.2948	2.3743	2.2429	2.1452	2.4623	2.3412	1.9611	2.6508
H	0.3067	0.4407	2.7501	2.0792	2.4729	2.4550	0.3951	0.2801	2.2500	2.5603	2.7094	2.1352

	Number of clones	%
A_{405} > 2.2986	30	32.26%
1.9053 <= A_{405} <= 2.2986	25	26.88%
A_{405} < 1.9053	38	40.86%

Medium mutation rate (M)

	1	2	3	4	5	6	7	8	9	10	11	12
A	2.9413	1.7041	2.8003	0.3188	2.0968	1.9179	1.8900	2.0567	2.1583	0.2936	1.9112	0.9586
B	3.4955	2.2715	2.2402	0.9085	1.6259	1.9326	1.8537	2.0103	2.3166	0.4998	2.0768	2.2606
C	3.0368	2.2366	2.2430	1.9220	1.9788	1.9051	0.4894	0.4052	1.5941	2.3542	1.0336	2.0701
D	0.4167	2.1061	0.3117	0.3097	2.1195	2.2012	1.3002	2.3451	0.3137	2.3529	0.3059	0.9668
E	2.5154	2.2771	2.3350	0.3204	2.3090	1.9942	1.0736	2.8361	0.2620	2.2218	0.7065	2.0495
F	0.6966	0.5466	1.4318	2.0967	0.7458	2.4137	2.3783	2.4722	2.1796	1.2112	2.3013	1.8278
G	3.0056	0.2827	0.4645	2.1505	2.2956	0.3041	2.1294	2.3702	2.1397	1.9913	2.2037	2.0822
H	1.0252	3.3457	0.3527	2.9660	2.7675	2.2693	2.2481	1.1113	0.3728	2.4342	0.3040	2.3509

	Number of clones	%
A_{405} > 2.2986	23	24.73%
1.9053 <= A_{405} <= 2.2986	31	33.33%
A_{405} < 1.9053	39	41.94%

High mutation rate (H)

	1	2	3	4	5	6	7	8	9	10	11	12
A	3.0989	0.3218	2.5087	0.2763	0.2729	0.8756	0.6360	0.8572	0.2939	2.0422	2.1291	3.1921
B	1.3540	1.9983	2.2634	0.2825	1.6362	2.3447	0.6796	1.4289	0.2832	0.2479	0.3864	1.8909
C	2.4528	0.2976	0.3250	0.2685	1.6409	0.2706	0.8066	0.2974	0.3133	0.2720	0.2639	1.5140
D	0.4309	0.4324	0.2825	0.3668	2.0345	0.2877	2.2517	0.4252	0.6076	1.6695	0.3025	2.1835
E	0.8755	0.2905	0.3471	0.2715	0.2760	1.9196	0.4361	0.2531	0.2702	1.8669	2.5279	0.2813
F	3.7770	0.4291	0.3700	1.5488	0.2625	0.2759	0.2663	1.8647	1.7033	0.4947	0.4564	0.3032
G	0.4936	2.3448	3.3184	0.3492	0.3427	0.2716	0.3400	0.3280	0.4657	0.2926	2.1346	0.6533
H	3.6444	3.1630	0.3281	0.4461	3.4670	0.3463	3.2322	0.6446	3.2634	0.3362	0.4180	3.2709

	Number of clones	%
A_{405} > 2.2986	15	16.13%
1.9053 <= A_{405} <= 2.2986	6	6.45%
A_{405} < 1.9053	72	77.42%

Figure S6: Sequence chain view of the structure of DyP4 F194Y variant (PDB 6FSK). Red triangles indicate the positions of missense mutations found in 4D4 variant. Other highlighted residues include the following: (i) proximal histidine (magenta dot) and arpartic acid (red dot), and (ii) distal arginine (cyan dot) and aspartic acid (orange dot), with the latter forming part of the GXXDG motif.

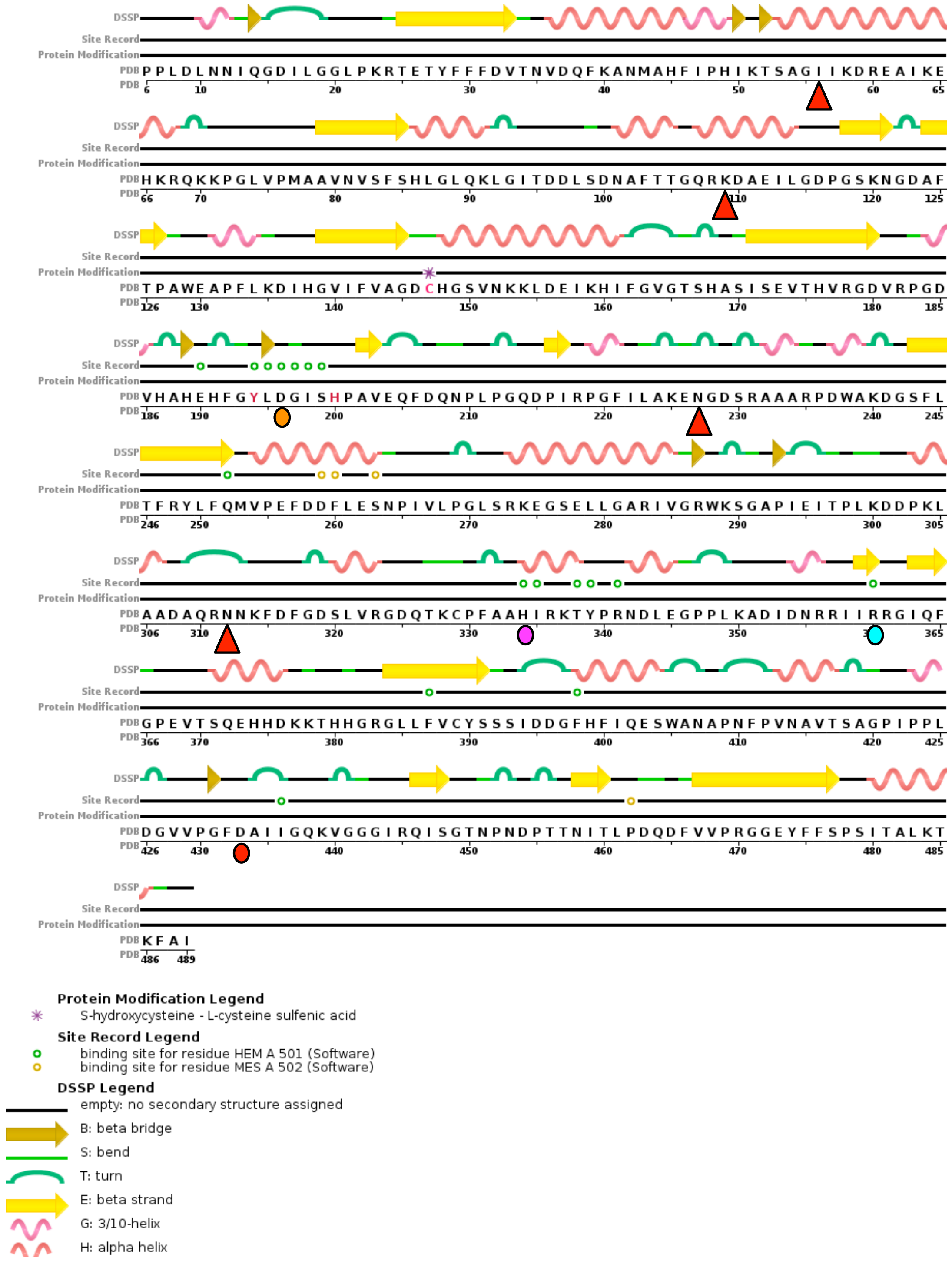

Figure S7: Cell pellets of WT, 3F6, 4D4, OsmY-WT, OsmY-3F6 and OsmY-4D4, after protein expression in 2×TY medium.

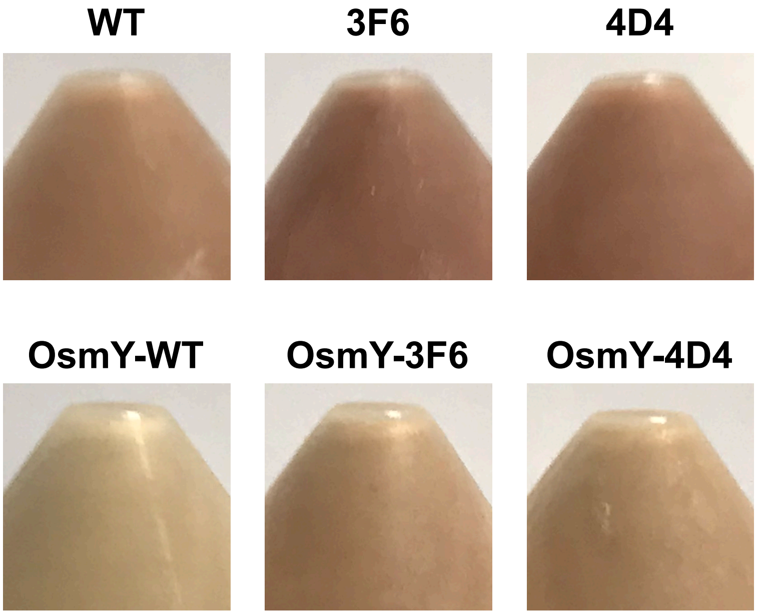

Figure S8: SDS-PAGE of the protein extract and the purified protein of DyP4 WT and its 3F6 and 4D4 variants.

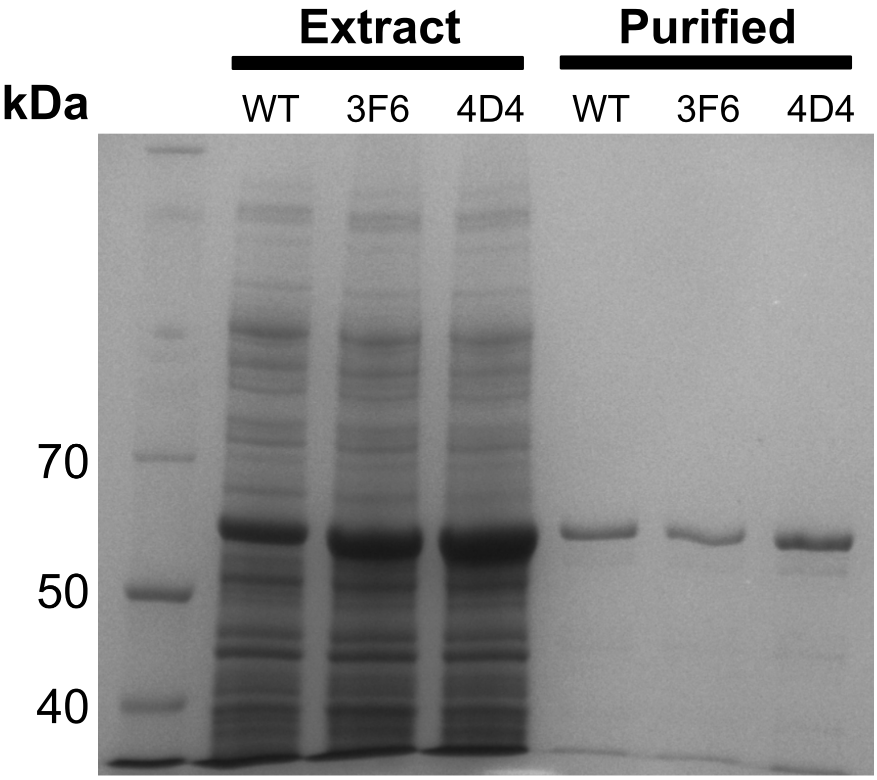

Figure S9: SDS-PAGE of acetone-precipitated OsmY-WT, OsmY-3F6 and OsmY-4D4. Purified WT was included as reference.

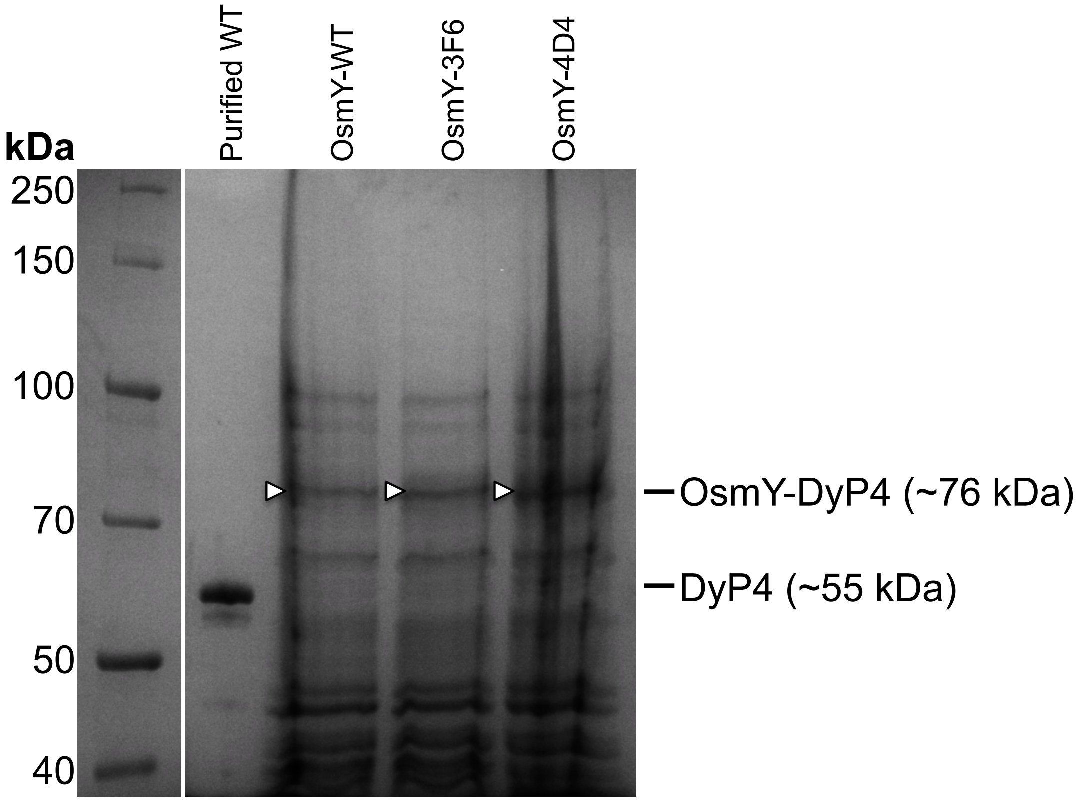

Figure S10: Chromatogram of the size exclusion chromatographic (SEC) step of OsmY-DyP4 purified from cell pellet. The inset shows the SDS-PAGE of cell extract and fractions 2, 4, 5, 6 and 7.

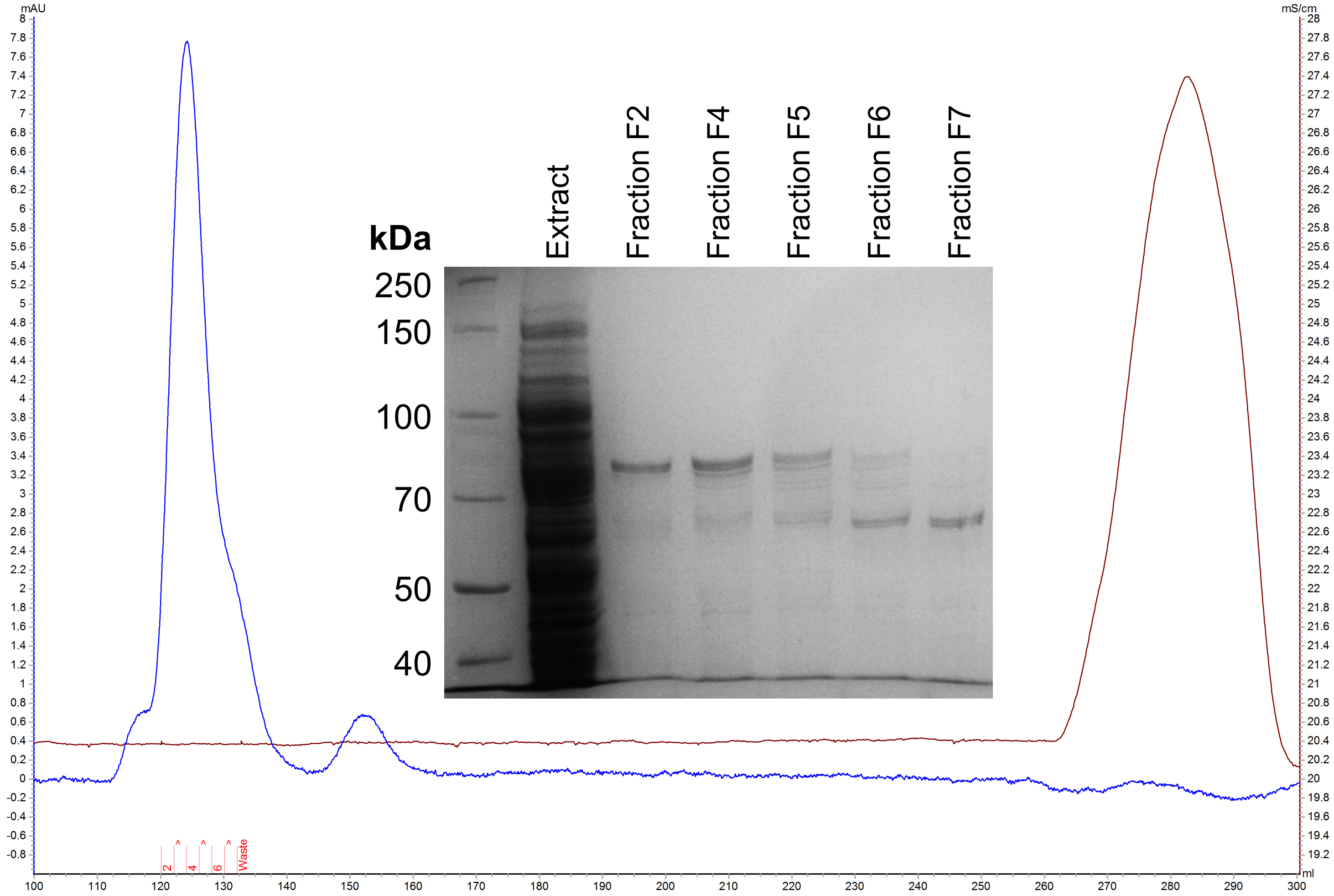

Supplement: Supplementary file 1 — Additional file 1. Supplementary material. [file 40643_2019_255_MOESM1_ESM.pdf]
